# *Ex vivo* evaluation of tumor cell specific drug responses in malignant pleural effusions

**DOI:** 10.18632/oncotarget.20889

**Published:** 2017-09-15

**Authors:** Carl-Olof Hillerdal, Rita Ötvös, Tünde Szatmári, Sulaf Abd Own, Gunnar Hillerdal, Åsa-Lena Dackland, Katalin Dobra, Anders Hjerpe

**Affiliations:** ^1^ Karolinska Institutet, Department of Laboratory Medicine, Division of Pathology, Karolinska University Hospital, SE-141 86 Stockholm, Sweden; ^2^ Gävle Hospital, Department of Lung Medicine, 803 24 Gävle, Sweden

**Keywords:** personalized medicine, drug sensitivity testing, malignant mesothelioma, lung cancer, pleural effusions

## Abstract

The effect of chemotherapy may be improved by combining the most effective drugs based on testing the sensitivity of the individual tumor *ex vivo*. Such estimations of tumor cells from effusions have so far not been implemented in the clinical routine as a basis for individualized choice of therapy. One obstacle for such analyses is the admixture of benign cells that might obscure the results. In this paper we test and compare two ways of performing the analysis specifically on tumor cells. First we enrich the tumor cells, using antibody labeled magnetic separation, and measure the effects of subsequent drug exposure with the metabolic activity assays WST-1 and alamar blue. The second way of estimating drug effects specifically on tumor cells employs multi parameter flow cytometry, measuring apoptosis with the propidium iodide / AnnexinV technique and, particularly for pemetrexed, possible effects on cell cycle progression in immunologically identified tumor cells. The two techniques produce similar results, indicating a possible use in personalized medicine. The possible predictive role of the analysis remains to be shown.

## INTRODUCTION

Around 80 % of all patients with a malignant mesothelioma (MM) have an associated effusion in the serosal cavities, most often in the pleura but also in the pericardium and peritoneum. Spread of a tumor to these serous cavities is also seen from lung adenocarcinoma (LAC) [1, 2] and from other primary tumor sites. The effusion is drained to relieve symptoms such as dyspnea, thus providing the first material available for diagnosis [3–5]. Depending on previous exposures to asbestos or asbestos like minerals in the population a highly variable proportion of cancers in the pleura are primary MMs. In most centers, however, the vast majority of pleural malignancies are metastatic adenocarcinomas, most of them originating from a primary tumor in the lung, breast, ovary or gastrointestinal tract.

The prognosis for patients with a malignant pleural effusion (MPE) is poor. In a study the median overall survival (OS) was 9.5 months for MM [6] and 5 months for LAC with MPE [2, 7, 8]. Curative surgical interventions are rarely possible for patients with such malignant involvement of the serous cavities. The main option to improve survival of these patients is chemotherapy.

The principal chemotherapeutic agents used for treatment of MPE are platinum analogues in combination with pemetrexed (PC), taxanes or gemcitabine [9]. For both MM and squamous carcinomas of the lung, pemetrexed in combination with platinum analogues are the most often used first line treatment [10, 11], while the combination of gemcitabine and platinum analogues (GC) is a common first line treatment for LAC [12, 13], although whether these regiments constitutes optimal care is not uncontested [14]. Other options for the treatment of MM are vinorelbine as a single drug or in combination with gemcitabine (VG) or platinum analogues (VC) [15–17]. Another alternative is the combination of platinum analogues, liposomal doxorubicin and gemcitabine (CDG). While originally developed as a first line chemotherapy for patients with MM it is today often seen as a third line regimen [18]. In studies these regimens have shown response rates in the order of 30-40% [19, 20].

We have previously shown a high variability in drug sensitivity patterns, analyzing tumor cell isolates from effusions of MM and LAC patients [21, 22]. This suggests that the effect of treatment might be improved with the development of a personalized choice of drugs rather than the presently used standard protocols. Such attempts to determine the sensitivity of individual tumors to different drugs *in vitro* have been performed since long. While individual studies have had marked success for prediction [23–25], the analysis is not yet considered sufficiently valid for application in a clinical routine [26, 27].

When analyzing tumor cells in an effusion, a possible confounding factor is the highly variable admixture of benign cells. Most such non-malignant cells in the malignant effusion are lymphocytes and macrophages while in other cases benign mesothelial cells constitute a considerable portion. These cells can grow in short term culture but their ability to survive in culture is different from the corresponding cells in their natural tissue environment. The sensitivity of these cells to drug exposure may be different from that of the malignant cells; therefore obtained results might deviate from that of the tumor.

To assess the effects of drug exposure in short term cultures colorimetric assays are commonly used, measuring the proportion of viable cells after drug exposure. The advantages of these colorimetric assays are their speed and simplicity, allowing the test of multiple drugs at several concentrations in the same colorimetric reaction [28]. To overcome the problem with simultaneously present benign cells, the proportion of tumor cells can preferably be increased by some form of cell sorting, such as with the MACS-beads technique.

An alternative way of evaluating the effects of drugs is to demonstrate the development of apoptosis. One technique to demonstrate this is by the Annexin V / propidium iodide (PI) technique. Thus flourochrome tagged Annexin V added together with PI allows the detection of early and late apoptosis, using flow cytometry (FACS) [29, 30]. A particular advantage with FACS is that it allows the analysis of individual cells within a population and that these distribution characteristics can be obtained specifically on tumor cells without previous enrichment of these cells. With FACS it is also possible to demonstrate cells in early S-phase as an indication of cell cycle arrest and/or appearance of apoptotic bodies prior to the G1 peak, both indicating an effect of drugs like pemetrexed [31].

The aim of this study was to find means to measure drug sensitivities specifically in tumor cells isolated from effusions, also in samples dominated by benign cells. We analyze the sensitivity to the standard drugs: carboplatin/cisplatin, pemetrexed, doxorubicin and gemcitabine in cells from malignant effusions. The effects of both single drugs and their combinations are compared. Two alternative ways to make the analysis tumor cell specific are tested; either colorimetric assays based on metabolic activity after enrichment of tumor cells based on MACS-bead technology or multiparameter FACS-based analysis of Annexin V and PI reactivity, where size separation complemented with tumor specific antibodies gives tumor specificity. In both instances, we present the drugs toxic effect on the tumor cells as SI, Survival Index. SI is defined as the proportion of viable cells remaining in the treated sample as compared to an untreated control. For pemetrexed and the platinum analogues we also perform FACS-based cell cycle distribution assays, as the effects sometimes cannot be detected by other methods. For these assays, we instead of SI compare fold change of the proportion of cells in S-phase, comparing the treated samples with the untreated controls.

## RESULTS

### Titration of working drug concentrations

Following 48 hours’ exposure, the toxicity of the drugs was assessed as SI, Survival Index. SI was calculated as absorbance (WST-1) or emission (alamar blue) / proportion of viable cells (FACS; viable cells are cells non-labelled by PI and / or annexin V) of sample divided by absorbance / emission / proportion of viable cells of an untreated control. When relevant, the method that was used to measure SI is denoted by SI_COLO_, for colorimetric assays, or SI_FACS_, for the annexin V / PI FACS based assay.

**Figure 1:**
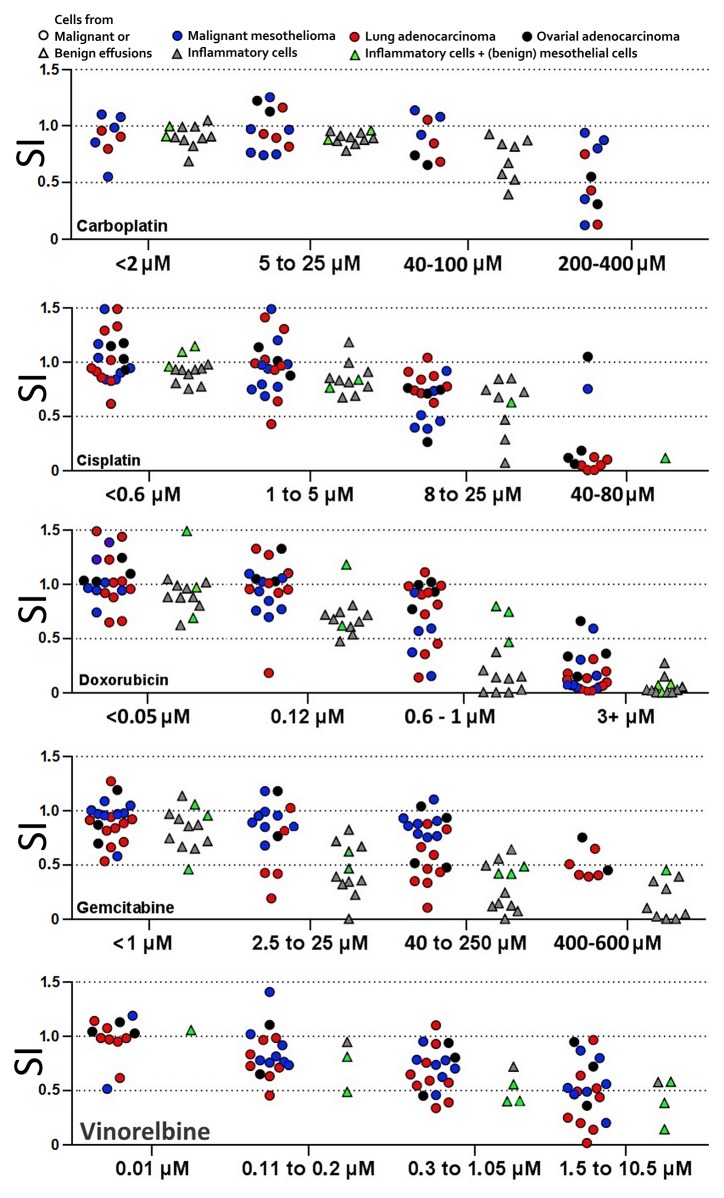
Titration to find drug concentrations that will distinguish more and less sensitive tumor cell isolates Cells from benign effusions kept under culture conditions were also sensitive to the drugs at these working concentrations. For each drug, concentrations with similar ratio of isolates reaching below SI 0.75, 0.5 and 0.25 respectively were designated to the same interval. Each patient isolate is represented only once in each interval. SI = survival index (WST-1 absorption of sample / ctrl).

When comparing patient isolates with each other’s, using to low concentrations is suboptimal, as most isolates will show resistance, and using to high concentrations is likewise suboptimal, as most isolates will show full toxicity. Thus, concentrations that will show an effect for most isolates are best suited to investigate differences in drug response between patients. Such concentrations are denoted ‘working’ concentrations. Combination experiments in particular depend on using consistent concentrations, as comparisons between isolates otherwise becomes difficult. The first 24 isolates were therefore tested against 2-4 concentrations of each drug, using previously determined “clinical” concentrations [21, 22] as a starting point and expanding outward to establish optimal working concentrations for each drug. However, this study also assessed FACS, combination experiments and cell cycle experiments in parallel with this titration. Therefore, working concentrations were initially substituted by 30% maximal effective concentration (EC30), of cell line Stav-AB and M-14-K, based on the toxicity seen with the bimonthly drug efficiency verification experiments. After the 24 first isolates, cell line EC30 were then supplemented with the working concentrations (see Table 1). The metabolic activity was in some cases found to be higher in drug exposed cells than in their controls. Such hyperactivity may indicate subsequent apoptosis (see Supplementary Figure 1) [32]. Effects of pemetrexed could not be determined using colorimetric assays after 48 hours’ drug exposure, even when using relatively high drug concentrations. Instead, the clinical concentration of 50 μM and a 5x higher concentration were used, tracing the effect by cell cycle analysis. The working concentrations could demonstrate differences between the different primary tumors, although the numbers in each tumor group is too low to generalize to tumor type. When tested on benign cells under these culture conditions, the drugs generally had stronger effects than was seen with the malignant cells (Figure 1).

**Table 1:** The working concentrations and cell line EC30 concentrations used for combinations to visualize possible synergism

	Carboplatin	Cisplatin	Gemcitabine	Doxorubicin	Pemetrexed	Vinorelbine
**Working concentration**	25 μM	8.0 μM	50 μM	0.024 μM	250 μM	1.05 μM
**Cell line EC30**	100 μM	2.0 μM	5 μM	0.6 μM	50 μM	10.5 μM

These concentrations were also used for FACS and spheroid experiments. The working concentrations were titrated using the first 24 isolates, during which the cell line EC30 concentrations served as substitute working concentrations.

Analysis of variance, performed independently for the three methods WST-1, alamar blue and FACS Annexin V / PI, showed that a difference in SI of 0.14 for alamar blue, 0.26 for WST-1 and 0.17 for FACS indicated significant difference. This distance is henceforth referred to as the discriminatory difference.

### Comparing drug effect as measured by colorimetric assays and FACS

**Figure 2:**
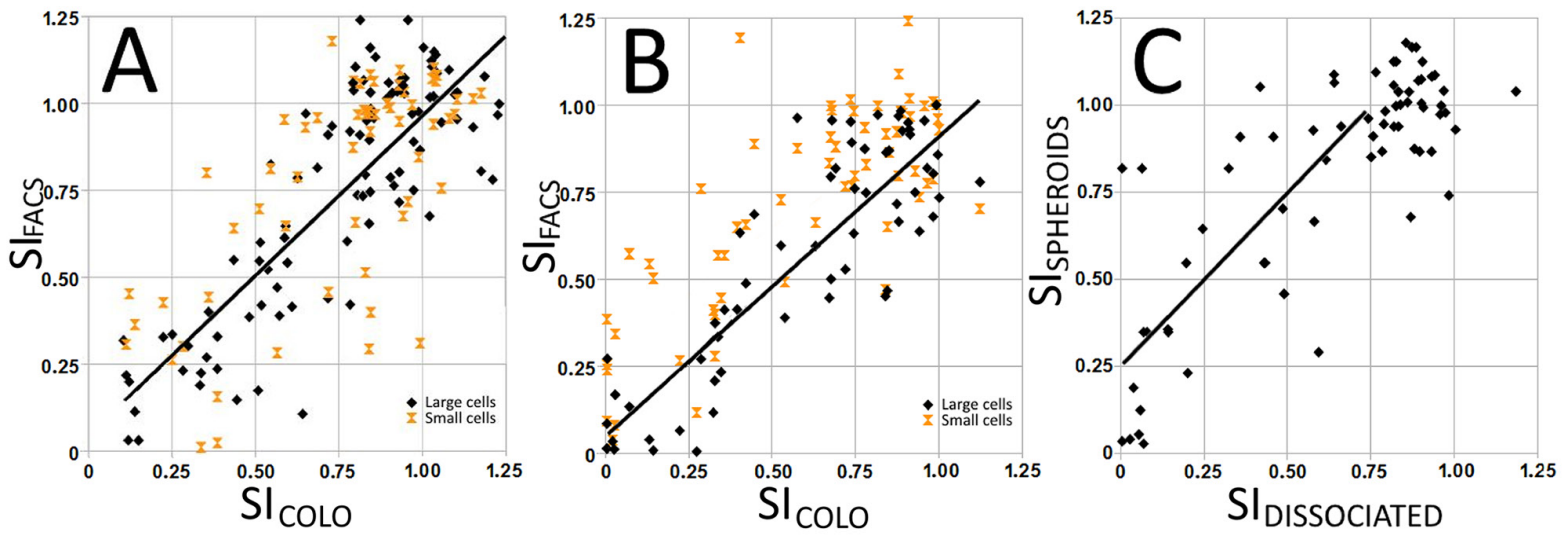
Correlation between survival index SI_COLO_ (metabolic activity) and SI_FACS_ (proportion of non-apoptotic cells) in cell isolates from malignant **(A)** and benign **(B)** effusions grown on hydrophilic plates. SI_COLO_ and SI_FACS_ correlated for large cells, while there was no such correlation for small cells, indicating that their contribution to SI was negligible. When grown on a hydrophobic support that promotes the formation of speroids **(C)**, the obtained survival index shows less drug effect compared to cells grown on hydrophilic plates.

Parallel (WST-1) or serial (alamar blue) and FACS Annexin V / PI measurements showed a statistically significant linear correlation (p < 0.0001; Figure 2). The slope of 1.05 for malignant and 1.16 for benign cells indicates that the two analyses measure the same biological effect at 48 hours. When the linear correlation is measured outside the metabolic hyperactivity region (SI_COLO_ > 0.75), the slopes (1.09 and 0.97, respectively) indicate an even closer correlation between the ways of measuring the drug effects. There was a tendency that the effects of platinum drugs were more pronounced when measured by FACS than was seen by the colorimetrical activities, while an opposite trend was seen with vinorelbine. Cells defined by FACS as small – mainly inflammatory cells – had significantly less contribution to total metabolic activity than large cells and will thus interfere less when measuring drug effects.

When cells were seeded on hydrophobic and hydrophilic plates, the former may induce spheroids artificially by hampering adhesion of cells to the support [33]. On the other hand, cells seeded on hydrophilic support adhere and form less cell-cell contacts. Different sensitivities were obtained when keeping the cells on these different supports. When grown to promote spheroid formation, the isolates became generally more resistant with an average increase in SI_FACS_ and SI_COLO_ values of 0.25 (Figure 2C).

### Accuracy of tumor cell specific FACS analysis

**Figure 3:**
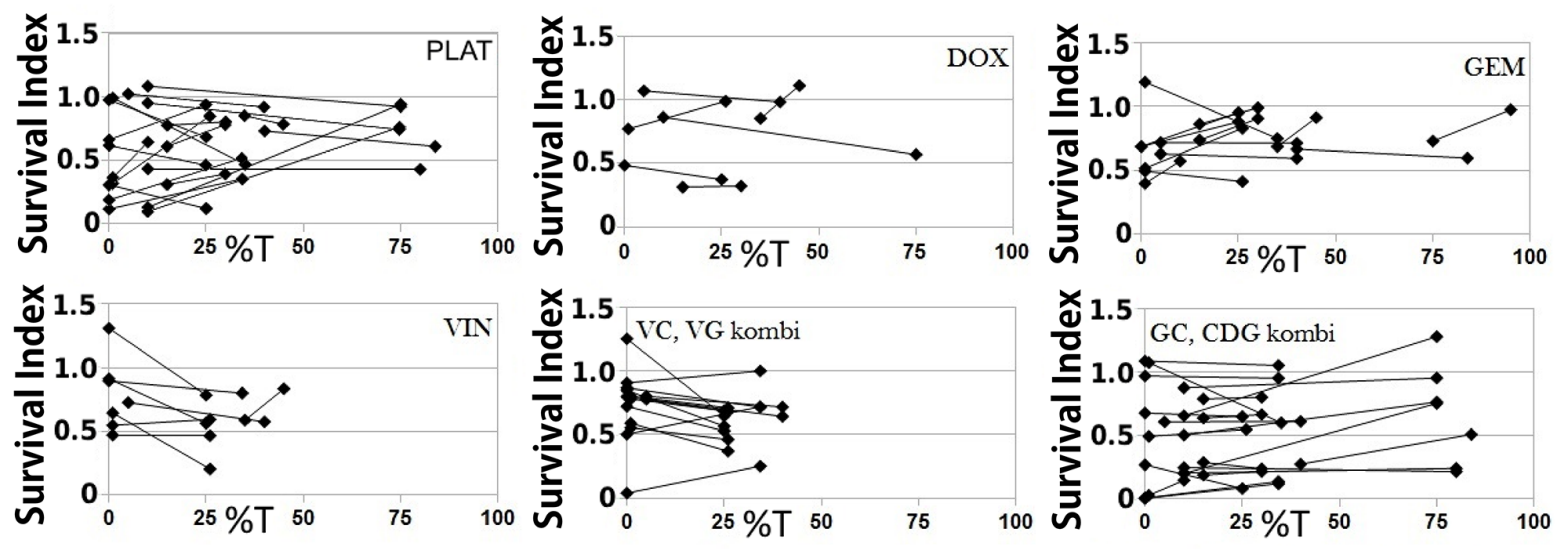
When tumor cells are enriched by antibody labeled magnetic separation, the obtained survival index (SI) is often altered, demonstrating the impact of benign cells The data pairs represent SI values obtained with the same cell isolate, comparing preparations depleted and enriched of tumor cells by the MACS beads technique. %T = tumor cell proportion.

Enrichment was generally attempted on preparations with around 50% tumor cells or less. In most cases the MACS-beads technique combined with filters increased the proportion of tumor cells at least twofold. The yield from viscous and / or turbid effusions was generally worse than from clear effusions. When comparing enriched and depleted preparations, obtained SI_COLO_ values varied considerably (Figure 3), highlighting the need for high proportions of tumor cells in the colorimetric assay. With FACS, on the other hand, the readouts are tumor cell specific, using size gating in combination with antibody labeling (Supplementary Figure 2). Tumor cell proportions estimated from routine immunocytochemistry and from FACS analysis were highly concordant. Comparing tumor cell specific drug sensitivity data determined by FACS and corresponding non-specific data from colorimetric assays also shows the need for tumor specific analysis. Differences in SI_FACS_ (tumor population) and SI_FACS_ (large cell population) were always below the discriminatory distance for isolates with 54% or more tumor cells. However, theoretical calculation of the macrophage influence on the total SI value based on obtained SI_FACS_ values for the respective tumor and macrophage populations varying the tumor cell proportion indicate that 75% tumor cells are needed to ensure that the difference remains below a discriminatory distance of 0.17. This means that when the proportion of tumor cells exceeded 75%, the influence of the benign cell admixture never caused a statistically significant change of obtained SI value.

**Figure 4:**
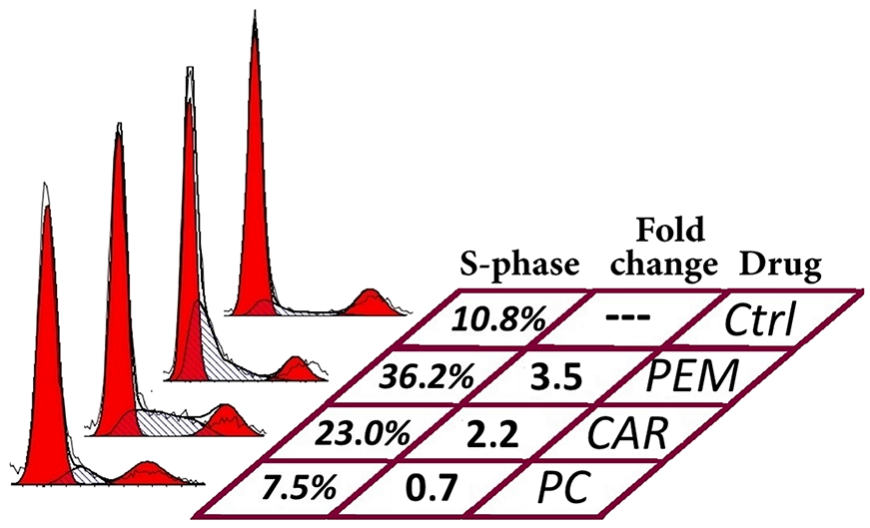
Effects of pemetrexed (PEM), carboplatin (CAR) and their combination (PC) on cell cycle distribution in a cell isolate containing malignant mesothelioma cells Both drugs cause increased proportion of S-phase cells when given individually, while they together seem to block S-phase entry.

As mentioned above a 48 hours’ exposure to pemetrexed at 50 and 250 μM concentrations will almost never induce apoptosis. However, by analyzing cell cycle phases, an effect of pemetrexed can at this time be seen as an increase in early S-phase while the G2/M peak eventually diminish. Like pemetrexed, carboplatin is also able to induce S-phase arrest without eliciting apoptosis response within the 48 hours’ of exposure (Figure 4). Furthermore, carboplatin activity as measured by SI at 48 h was in 5/27 isolates hampered by simultaneously given pemetrexed (Supplementary Figure 3). This was evident regardless if demonstrated by FACS or colorimetrically.

### Drug combinations and correlation to clinical outcome

When testing the drugs in combination, the sensitivity patterns differed from those obtained from testing the drugs one-by-one. In some cases the toxicity seen with combinations were mainly due to one of the included components and in others the combination had profound effects even when all drugs showed a complete resistance when tested individually (Table 2).

**Table 2:** Drugs in combination may indicate sensitivity also when no effect is seen when tested individually

		CDG	CDg	CdG	Cdg	cDG	cDg	cdG	cdg	CisDG	cisdg
MM	Exp	**1.00**	**1.00**	**1.00**	**1.00**	**1.00**	**1.00**	**1.00**	**1.00**	**1.00**	**1.00**
	Obt	**0.43**	**0.75**	**0.61**	**0.74**	**0.89**	**0.83**	**1.05**	**0.93**	**0.22**	**0.70**
LAC	Exp	**0.19**	**0.54**	**0.19**	**0.54**	**0.19**	**0.54**	**0.19**	**0.54**	**0.19**	**0.54**
	Obt	**0.05**	**0.36**	**0.05**	**0.52**	**0.22**	**0.48**	**0.21**	**0.48**	**0.05**	**0.55**
OAC#1	Exp	**0.57**	**0.54**	**0.57**	**0.54**	**0.76**	**0.72**	**0.76**	**0.72**	**0.58**	**0.73**
	Obt	**0.12**	**0.17**	**0.12**	**0.15**	**0.73**	**0.61**	**0.65**	**0.69**	**0.05**	**0.43**
OAC#2	Exp	**0.66**	**0.66**	**0.66**	**0.66**	**1.00**	**1.00**	**1.00**	**1.00**	**0.27**	**0.88**
	Obt	**0.46**	**0.50**	**0.41**	**0.46**	**1.20**	**0.80**	**1.12**	**0.95**	**0.20**	**0.71**

The theoretical additive effect (SI_COLO_) was calculated for each combination (Exp surv) and compared to the actual reading (Obt surv). This difference often exceeded the discriminatory distance (0.26 for WST-1). (C=carboplatin, D=doxorubicin, G=gemcitabine, Cis=cisplatin; capital letter indicate that the concentration that was highest of working or cell line EC30 concentration was used, while lower letter indicate the lower concentration (see Table 1); MM=malignant mesothelioma, LAC=lung adenocarcinoma, OAC=ovarian adenocarcinoma).

Predictions were based on stratifying the *in vitro* results; the upper half of the 50% most affected isolates was considered strongly indicative of sensitivity and the lower half weakly indicative of sensitivity. All other results were considered non-responsive.

Response assessment based on the clinical evaluation, which takes mainly in consideration the result of radiological investigation, started 3 months after initiation of the treatment. Size of the tumor, appearance of new infiltrates, presence of metastases and the general condition of the patients are parameters which have a big role in the assessment.

There were six MM samples where tumor specific cell cycle analyses had been performed, the patients had been administered the carboplatin and pemetrexed combination and the response of the given treatment had been evaluated. Two isolates were predicted to have a strong response, (2.5 fold increase in S-phase, comparing treated and untreated sample) and both of those had stable disease. Three isolates were predicted to have a weak response (1,5 fold increase of S-phase, comparing treated and untreated sample). One patient showed stable disease, one patient showed progressive disease and the last patient died before the evaluation date. One isolate was predicted to have no response, and this patient had progressive disease. Three further isolates with a high content of tumor cells were only evaluated colorimetrically. Two of these MMs showed response with SI_COLO_ exceeding the discriminatory difference from non-responders. Both were stable disease while the third non-responding case showed progressive disease. Thus, the prediction based on the analysis of tumor cells in effusions correlated to clinical outcome in 7 out of 9 cases.

## DISCUSSION

Through the years there have been extensive effort to measure chemosensitivity of tumor cells, in order to predict drug responses and to individualize therapy for individual patients (for review see [34–36]). The analysis of primary tumor cells derived from effusions is one possibility for such drug sensitivity testing. Such effusions are mainly obtained from the pleura but also from the pericardium and the peritoneum. As these fluids are drained to reduce patient discomfort, they are often the first material available for morphological diagnosis, using cytopathology. When the fluids are sent fresh to the laboratory, they provide an excellent source of primary tumor material that can be grown in short term cultures for diagnostic as well as prognostic testing.

These effusions always contain variable amounts of malignant cells with an admixture of different benign cells mainly in the form of macrophages, inflammatory cells (granulocytes, lymphocytes) and benign, sometimes reactive mesothelial cells. These benign cells are not immortalized, and react unpredictably under culture conditions. Still some of these benign cells have an extreme growth rate *in vitro*, and can actually outcompete the tumor cell component, while others don’t attach or grow. The obtained sensitivities to drugs sometimes differ significantly, when comparing SI_COLO_-values of the same isolates before and after MACS-based enrichment of tumor cells. When the tumor cell content exceeds 75%, this effect of benign cell admixture seemed to influence the results less, allowing the determination to be based on colorimetrical analysis in such cases. With lower proportion of tumor cells, however, a more tumor specific analytical concept is advised. This is also supported by the demonstrated linear correlation between results from colorimetric analysis and tumor cell specific FACS.

However, a high content of tumor cells in the effusion is associated with a more advanced stage of the disease, with less chance of response to treatment. It is therefore of major importance that the predictive test can be done also with few tumor cells present. In these cases the multi-parameter FACS analysis provides an opportunity to evaluate drug effects specifically on the tumor cells. Tumor cells should then be defined by immunological tumor markers, which also can be complemented with cell size. A minimum of 400 tumor cell registrations should be measured to warrant a confidence interval of obtained SI_FACS_ value within ±0,05.

Such a FACS-based population distinction has two main advantages over measuring colorimetric assays. First, the proportion of tumor cells was often not increased enough by the MACS-bead procedure and, secondly, FACS allowed the tumor cell specific analysis also with all original cell populations present during drug exposure. It can be speculated that such drug exposure together with the entire benign cell populations might better reflect the possible drug effects in the patient. One such influencing factor may be the admixture of macrophages, where presence of tumor cells alters the drug sensitivity of these macrophages. These macrophages may also affect tumor behavior [37]. Still, the FACS analysis is more resource demanding and may preferably be used in effusions with less than 75% tumor cells, equivalent results being obtained with colorimetrical assays with only spare benign cells present. Magnetic- or filter separation prior to colorimetric assays presents an alternative to FACS for isolates of below 75% tumor proportion down to around 30%, as this method can increase the tumor proportion twofold. However, this method becomes very labor intense if a large quantity of cells is required.

As the standard of care drugs elicited insufficient responses after a 48 hours’ exposure, the drug concentration ranges were optimized to distinguish more and less sensitive isolates. The short time culture approach is more practical for large drug screenings, since there is no need to exchange the cell culture medium during exposure. With such a short time for drug exposure, it is necessary to increase drug concentrations, in some cases substantially, compared to assumed tissue concentrations during treatment. When the working concentrations (see Table 1) were used in combinations (GC, VG and VG), SI less than 50% was obtained in roughly half of the tested isolates (see Supplementary Figure 3B). Pemetrexed, used as a single drug, similarly doubled the proportion of cells in S-phase arrest for approximately half of the tested isolates (see Supplementary Figure 3C). This is comparable to the expected overall clinical response of 30-40%. It must, however, be noted that some of the working concentrations were higher than the estimated tumor cell exposure in patients. Thus, the predictive value of obtained drug effects is difficult to appraise without correlation to clinical outcome.

We were able to find working concentrations for all drugs using colorimetric assays, except for pemetrexed. We have previously reported that the pemetrexed effect at 48 hours is best measured as S-phase arrest and we now confirm that this is equally true for primary patient material (Supplementary Figure 3C). During this short exposure there seemed to be antagonistic effects from platinum drugs and pemetrexed (Supplementary Figure 3A, Figure 4); for several cell isolates, the effect of platinum drugs as singles was higher than the effect of platinum drugs in combination with pemetrexed. It may well be that during this short exposure, the pemetrexed induced S-phase arrest interfere with drugs targeting replication, in this case delaying the DNA-damaging effect of platinum. When prolonging the exposure to 72 h, the combined effect was instead synergistic (data not shown). However, the complicated relationship of platinum and pemetrexed warrants caution when predicting drug sensitivity.

It is well known the tumor cells react differently to drugs when dissociated and when in groups with established cell-cell contacts [38–41]. Tumor cell groups may occur naturally in the malignant effusion, in fact, presence of such groups indicate the malignant condition. These groups may contain both tumor cells and tumor cell stroma. Similar so called spheroids can be induced in culture by preventing cell attachment, as is seen with the hanging drop technique or by growing them on a hydrophobic surface, although these groups are devoid of stroma. In accordance with previous findings, drug sensitivity decreased, comparing spheroid-like aggregates and monolayer cultures of the same isolate [42, 43]. Thus, further optimizations of drug concentrations are necessary if drug sensitivity testing is performed on isolates grown under spheroid-forming conditions.

When tested *in vitro*, the various tumor isolates responded differently to the tested drugs and their combinations. Considering the fact that response rates to today’s standard of care drug regiments is only in the order of 30-40%, this indicates that a predictive *ex vivo* test can be a basis for an individualized choice of therapy that improves the effect of treatment. Information on responses to given chemotherapy is here so far only available in a limited number of cases. There seem to be a correlation between the prediction based on the *ex vivo* testing and the actual clinical outcome of treatment, but the number of cases is far too small for a valid evaluation.

To summarize, tumor cells isolated from effusions offer a possibility to perform predictive analyses as a basis for individualized choice of therapy. Consideration must then be given to the presence of benign cells and the importance of making the analysis tumor cell specific. Tests should then also include exposures to drugs in combination, as a combinatory effect might be profoundly different from that of drugs as singles. The present drug profiling data must, however, be validated against patient outcome in larger series.

## MATERIALS AND METHODS

### Effusions and patient characteristics

This study includes primary cells isolated from serous effusions from patients with malignant or benign diagnoses. The effusions were received from the diagnostic routine at the Department of Pathology and Cytology, Karolinska University Hospital in Huddinge, Sweden. All diagnoses were confirmed at subsequent follow up. Informed consent was obtained as approved by the regional ethics committee. Altogether, 39 malignant effusions -13 malignant mesotheliomas (MM); 12 lung adenocarcinomas (LAC); 9 ovarian adenocarcinomas (OAC) and 5 adenocarcinomas of other origin (ADCA) were analyzed.

Furthermore, we analyzed the cells from 12 benign effusions, also from diagnostic routine, to evaluate effects on benign cells in the effusion. While with three of the isolates we could utilize all of our methods, for most cases the amount of tumor cells in the effusion limited the number of analyses that could be performed on the same cell isolate. In total, the effects of drug exposures were tested with colorimetric assay in all 39 malignant isolates, while FACS Annexin V / PI analysis could be evaluated in 17 cell isolates. Working concentrations, separating more and less sensitive cell isolates after 48 hours’ exposure, were titrated using 24 isolates. Effects of drug combinations were compared using 32 samples while S-phase arrest was studied in 13 malignant cases.

### Isolation of primary cells and cell culture conditions

The effusions were obtained fresh in the laboratory and kept at 4°C until assay. Cells were isolated from the effusions by centrifugation first at 300xg for 5 min (to avoid aggregation of cells) and, when necessary, followed by 1000xg for 5 min (for complete retrieval). Thus isolated cells were suspended in Iscove's modified Dulbecco's medium (IMDM, Sigma, Sweden), supplemented with 100 IU/mL penicillin (Sigma, Sweden), 100 μg/mL streptomycin (Sigma, Sweden), 0.2% gentamycin (Invitrogen, Sweden), 2 mM L-glutamine (Sigma, Sweden) and 20% Fetal Bovine Serum (FBS, Sigma, Sweden) and seeded in BD primaria cell culture flasks (Becton, Dickinson and Company, Sweden).

These cultures were kept at 37°C in a humidified atmosphere with 5% CO2. Effusions with heavy admixture of blood were hemolyzed in pre-warmed 1X BD Pharm Lyze (Becton, Dickinson and Company, Sweden) for 30 sec to 2 minutes followed by washing once with PBS before seeding as normal. M-14-K and STAV-AB human MM cell lines were grown in parallel for bimonthly verification of drug efficiency. These cell lines were grown in RPMI 1640 supplemented with 10% FBS for the M-14-K cell line and 10% human AB-serum for STAV-AB.

### Tumor cell enrichment

Depending on the composition of the individual cell isolates, various methods were used to improve the relative proportion of tumor cells. Culturing the cells overnight often yields two distinct populations, depending on their growth preference (adherent or supernatant). Following separation of adherent and supernatant populations, dissociated cells were either used simultaneously in FACS and colorimetric assays *or* further enriched utilizing antibody labeled magnetic beads separation (CD326 or CD45; MACS-beads, Miltenyi Biotec, Germany) followed by colorimetric assays.

Adherent tumor cell groups were digested with trypsin and collagenase for 20 min facilitating cell dissociation during culturing. These cells were then used as above. However, non-adherent tumor cell groups found in supernatant will generally not dissociate. Therefore, these cell groups were instead collected by filters (30 or 40 μm pore sizes).

Tumor proportions in fractions were determined using immunocytochemical (ICC) reactivity to BerEp4, Mesothelin, EMA and / or CD45 to support the evaluation of cell morphology.

### *Ex vivo* evaluation of drug sensitivity

To test the sensitivity to the different drugs, cells isolated from effusions were incubated in IMDM as above. Cisplatin, gemcitabine, pemetrexed (Alimta), vinorelbine, carboplatin and liposomal doxorubicin (Caelyx) were obtained from Hospira (Sweden), Lilly (Indianaplis, US), PharmaCoDane (Sweden), Actavis (Sweden) and Janssen (Sweden), respectively. The effects of a wide variety of drugs were tested at 48 hours, including previously determined clinically relevant concentrations [21]. The efficiencies of drugs were tested bimonthly with concentrations covering the linear dose response range. Preserved effects were evaluated with GraphPad Prism software (GraphPad Software, San Diego, CA). The effects of the clinical concentrations were often limited. Therefore, drugs were instead tested at the concentrations killing 30% of cells of tested cell lines (celline EC30) during the bimonthly drug efficiency verification experiments. In order to distinguish more and less sensitive tumor cell isolates we identified “working concentrations” in the region of a linear dose response after an incubation of only 48 hours. This titration was performed exclusively by colorimetric assays, testing the fractions with high proportion of tumor cells and using at least two concentrations for each drug. Twenty-four cell isolates were used for this titration. Their median proportion of tumor cells was 51%, three isolates containing less than 25% and 9 isolates with more than 75% tumor cells.

The primary cell cultures were first grown overnight at 37°C in 5% CO_2_ to allow adherence and then for another 48 hours following the addition of drugs. Tumor cell specificity was ensured in two different ways. First, tumor cells were enriched by antibody labeled magnetic separation prior to drug exposure. Cell cultures were then exposed to the above-mentioned 6 different drugs, in triplicates, and seeded in 96 well plates. The proportion of surviving cells was then determined using WST-1 colorimetric assay. To find suitable working concentrations, the drugs were preferably tested at four different concentrations.

The second way to monitor tumor specific drug effects was performed by multichannel flow cytometry analysis, measuring apoptosis in immunologically identified tumor cells. Cell cultures were exposed to drugs in duplicates in 24-well plates using the established working concentrations (Table 1). All FACS samples were either run in parallel using WST-1 or in serial using alamar blue [44].

The effects of drugs were assessed as SI colorimetrically by comparing the exposed and untreated control cells (SI_COLO_, absorption (WST-1) or emission (alamar blue) of sample / control). The effects of drugs were determined by adding the alamar blue reagent (ThermoFisher Scientific, Waltham, Massachusetts, USA) 90 min or WST-1 reagent (Roche, Basel, Schweiz) 2 h prior to the end of the 48 hours’ incubation. Using this colorimetrical analysis, SI_COLO_ was calculated as the ratio of treated cells to parallel untreated controls. The corresponding SI_FACS_ was calculated by comparing the proportion of non-apoptotic cells in treated samples to those of untreated controls.

WST-1 is a tetrazolium salt cleaved by an outer cell membrane enzyme dependent on NADPH/H+ and then yield a colored product that can be quantified spectrophotometrically at 450 nm with subtraction of background absorbance at 600 nm. Alamar blue (resazurin) spontaneously react with intracellular NADPH/H+, yielding a fluorescent product (excitation at 540 nm, emission at 590 nm). Thus, these colorimetric assays are based on metabolic activity. Hence, a decrease in absorption (WST-1) or emission (alamar blue) is often indicative of a decrease of metabolically active cells (apoptosis, failure to replicate), and parallel use of both assays following cytotoxic drug exposure results in near identical results (data not shown). However, note that a sharp increase in absorption or emission occasionally is seen prior to apoptosis (see Supplementary Figure 1) [32].

Duplicate FACS analyses were based on 5.000-20.000 cellular events being recorded for each run, using a Becton Dickinson FACS Calibur flow cytometer. Spheroids formed during culture were dissociated by vigorous pipetting prior to FACS analysis. Based on the statistic CI_95%_ = 1,96√(p(1-p)/N), a minimum of 400 tumor cell registrations was considered necessary for validity; with a SI_FACS_ of 0.5 the 95% confidence interval will then theoretically be smaller than 0.05. Labelling was performed on non-permeabilised cells, using APC flourochrome conjugated tumor marker antibodies (EpCAM (Becton, Dickinson and Company, Sweden) for adenocarcinomas and mesothelin (R&D Systems, Minneapolis, USA) or EMA (R&D Systems, Minneapolis, USA) for MM. Viability was evaluated as non-apoptotic cells detected in the lower left quadrant with Annexin V and PI (FITC Annexin V Apoptosis Detection Kit I, Becton, Dickinson and Company, Sweden). APC labelling for isolates from benign effusions were instead CD45 (Becton, Dickinson and Company, Sweden). FACS gates were set according to side scatter (SSC), forward scatter (FSC) and to the tumor or inflammatory cell specific immunoreactivity. Example (tumor specific) FACS readouts are shown in Supplementary Figure 2.

The emitted light from the APC fluorochrome was detected at an emission wavelength of 660 nm (excitation at 633 nm) and from Annexin V at 530 nm (excitation at 488 nm). Presence of PI was detected at 670 nm (excitation at 488 nm). Adjustments were made for doxorubicin auto fluorescence. The Annexin V negative/PI negative population represents unaffected viable cells, Annexin V positive/PI negative represents early apoptotic cells and Annexin V positive/PI positive represents late apoptotic cells. Signals in the PI channel without corresponding high Annexin V reactivity were considered to be disintegrating dead cells. SI using FACS and annexin-V / PI was calculated by comparing the proportion of non-apoptotic cells in treated samples to those of untreated controls (SI_FACS_, proportion of viable (non-annexin-V and non-PI reactive) cells in population of interest in sample / control. Population of interest might be small cells (T-cells) or large cells (macrophages, tumor cells and mesothelial cells) using FSC and SSC. Tumor cells or large benign cells can be separated by using tumor markers or inflammatory cell markers.

### Cell cycle analysis

Since the effects of 48 hours’ exposure to pemetrexed is (most often) not detected by neither colorimetric nor Annexin V/ PI (FACS) assays [31], the possible S-phase arrest was traced by analysis of the cell cycle. After exposure to pemetrexed or the combination of carboplatin and pemetrexed, the cell membranes were permeabilised by fixation in ice-cold 70% ethanol. The samples were then incubated at 37°C for 30 minutes in PBS containing 1% BSA and 50 μg/ml of PI. The distribution histograms obtained with FACS were then used to quantify the proportion of cells in G0/G1 phase, S phase and G2/M phase, respectively. The results were quantified using ModFit software.

### *In vitro* test using drug combinations

Combinations of drugs were tested, as described above, together with their component drugs as singles in order to discern combinatory effects. The following combinations were used: pemetrexed and carboplatin or cisplatin (PC), gemcitabine and carboplatin or cisplatin (GC), carboplatin or cisplatin together with liposomal doxorubicin and gemcitabine (CDG), vinorelbine and carboplatin or cisplatin (VC) and finally vinorelbine and gemcitabine (VG). Choice of concentrations is paramount to combination testing; if the concentrations are either too low or too high then combinatory effects can be masked. Therefore, the effects of drug combinations were better analyzed using two concentrations of each tested drug. Apart from the determined “working concentrations” also the EC30 concentrations for cell lines were used (Table 1).

### Analysis of drug sensitivity, using hydrophobic support

Totally 8 isolates (5 malignant and 3 benign/reactive) were seeded on hydrophobic plates (Greiner Cellstar hydrophobic, VWR). The reduced ability to attach to these surfaces will facilitate the formation of spheroid-like groups [33]. For comparison, these samples were always grown in parallel with cells in monolayer. Both cultures were exposed to drugs at the established working concentrations (Table 1). Twenty-four well plates were used to avoid uneven distribution of tumor cells within the same isolate.

### Correlation to clinical effect of given drugs

Responses to individual drugs were retrieved from patient journals in accordance with the ethical permit. Altogether 8 MM patients had been treated with drugs tested *in vitro* (all with Pemetrexed and Carboplatin)*,* allowing comparison of prediction and outcome of treatment. Responses were evaluated based on clinical evaluation, taking into consideration the radiologically determined size of the tumor, appearance of new lesions, presence of metastases and the general condition of the patients, comparing initial conditions with those 3 months after initiation of treatment. Predictions were stratified based on obtained sensitivity data, including both cell cycle data and cell survival index (SI). Obtained effects of given treatment had been classified as progressive disease (PD) or stable disease (SD) while none of them showed partial or complete response.

### Statistical analysis

Results were analyzed using GraphPad Prism for correlation coefficients. The null hypothesis of no difference was rejected at a 0.05 significance level. Cell cycles were analyzed using the Cell Wizard in ModFit 3.3. A discriminatory difference, the difference in SI_COLO_ and SI_FACS_, which statistically indicate a true difference, was calculated using analysis of variance. The discriminatory difference was used to determine whether combinatory effects were additive or synergistic, and to determine whether the presence of benign cells changed the drug sensitivity profile in a significant manner.

## SUPPLEMENTARY MATERIALS FIGURES


