# Supplementary material for: Ex vivo evaluation of tumor cell specific drug responses in malignant pleural effusions
Source: Oncotarget. 2017 Sep 15;8(47):82885–96. doi: 10.18632/oncotarget.20889 (PMC5669936; doi:10.18632/oncotarget.20889)
Supplement: Supplementary file 1 [file oncotarget-08-82885-s001.pdf]

Ex vivo evaluation of tumor cell specific drug responses in malignant pleural effusions

SUPPLEMENTARY MATERIALS

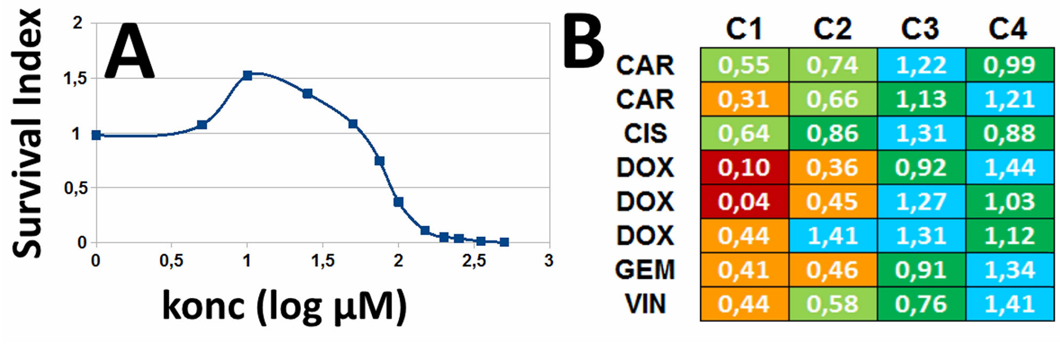

Supplementary Figure 1: Dose-response effect measured as WST-1 for M-14-K cell line treated with carboplatin **(A)**. Note the increase in metabolic activity at lower concentrations. Similar hyperactivity at lower concentrations was seen for all tested drugs (except pemetrexed) when applied to primary cell cultures **(B)**. C1 refers to the highest drug concentration and C4 to the lowest. Red denotes SI < 0.25, orange SI < 0.5, light green SI < 0.75, dark green resistance, and blue hyperactivity.

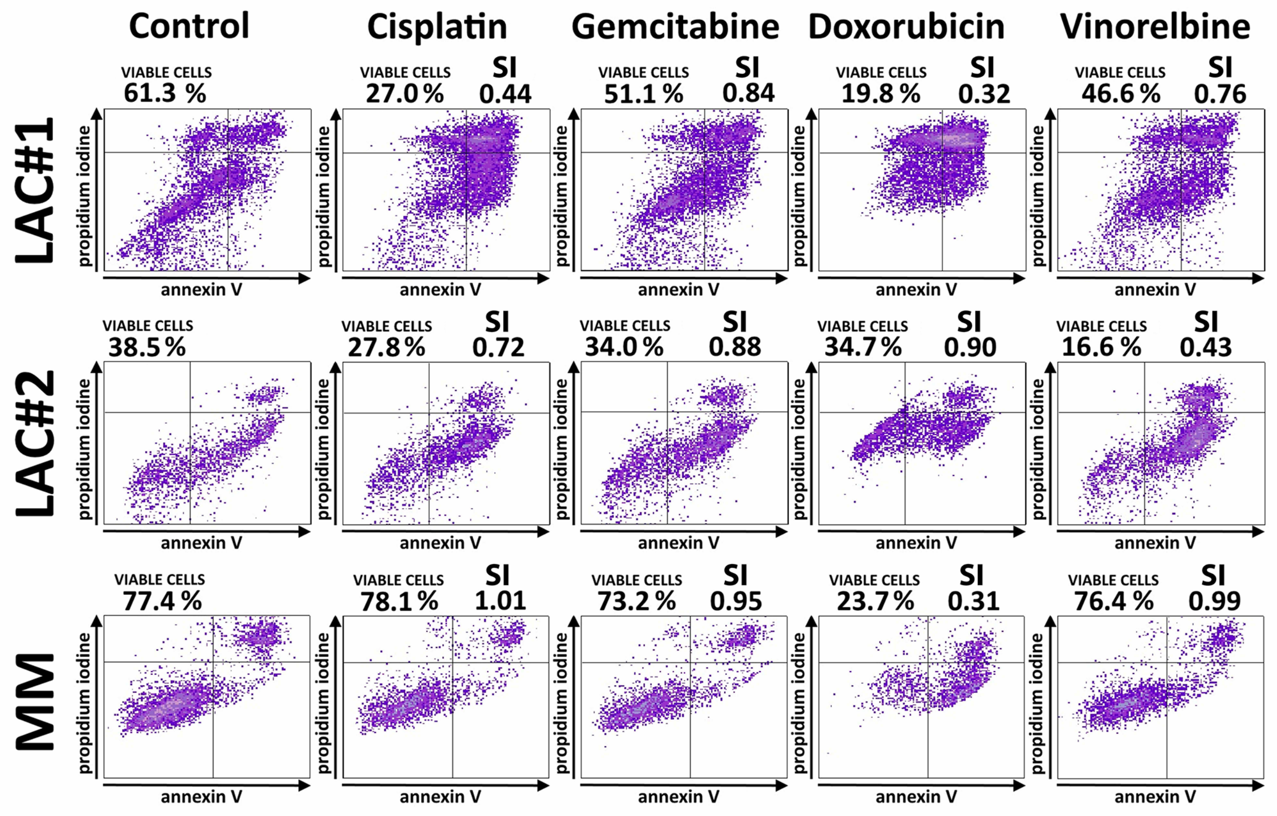

Supplementary Figure 2: Tumor cell specific apoptosis after drug exposure measured by FACS analysis. The lower-left quadrant represents non-apoptotic cells. Two lung adenocarcinoma (LAC) and one malignant mesothelioma (MM) illustrates natural variations in drug resistance. Note especially how LAC#2 is resistant to doxorubicin but sensitive to vinorelbine while the MM is sensitive to doxorubicin but resistant to vinorelbine.

A

Isolate	%T	PC	GC	CDG	VC	VG
MM#1	100*					
MM#2	80					
MM#2	15		SYN			
MM#4	5	ANT				
LAC#1	65,0					
LAC#2	45	SYN		SYN	SYN	
LAC#3	40					
LAC#4	35	ANT				
LAC#5	25					
LAC#6	5					
OAC#1	100*					
OAC#2	100*					
OAC#3	95					
OAC#4	50	SYN	SYN			
OAC#5	40	ANT	SYN			
OAC#6	35					
ADCA#1	100	ANT				
ADCA#2	40		SYN			
ADCA#3	20					
ADCA#4	<1					
ADCA#5	N/I	ANT	SYN			

* Tumor specificity using FACS

B

Isolate	%T	PC	GC	CDG	VC	VG
MM#5	75					
MM#6	35					
MM#7	30					
MM#8	30					
MM#9	25			SYN		
LAC#7	80					
LAC#8	75					
LAC#9	65					
OAC#7	90					SYN
OAC#8	80			SYN		
OAC#9	65					

Colour	
Dark green	SI >0.75
Yellow-green	SI <0.75
Orange	SI <0.50
Red	SI <0.25

C

	Pemetexed		Carboplatin		Combination	
Isolate	SI	S-phase	SI	S-phase	SI	S-phase
MM#2	1.04	3.5	1.04	2.2	1.09	0.7
MM#6	0.92	2.8	1.01	5.5	0.80	Inhibited
MM#8	1.03	2.1	1.14	2.4	1.01	Sub-G1
MM#4	1.19	1.5	0.80	Sub-G1	0.98	
MM#10	0.91	1.6	0.74			
MM#11	0.97	1	0.97			
LAC#6	0.92	3.7	0.79	2.4		
LAC#7	0.95	2.3	0.87		0.96	
LAC#2	0.95	1.4	0.84	2.4	0.78	Sub-G1
LAC#3	0.97	1.2	1.08		0.76	1.2
OAC#1	1.15	1.3	0.94	1	1.04	1.5
ADCA#4	0.92	1.4	0.37	0.6	0.45	

S-phase sample / ctrl	
Dark green	Fold change <1.5
Yellow-green	Fold change >1.5
Orange	Fold change >2.0
Red	Fold change >3.0
Light blue	No visible S-phase

Supplementary Figure 3: The combination experiments revealed variable sensitivity to different drug combinations. Resistance to one drug combination does not exclude sensitivity to alternative ones. SI_{COLO} was calculated as absorption (WST-1) or emission (alamar blue) sample/ ctrl, while SI_{FACS} (only three isolates, indicated by an asterix) was calculated as the proportion of viable (non-annexin and non-PI reactive cells) tumor cells, comparing sample with control. Working concentrations (see Table1) were used based on EC30 of cell lines **(A)**, and based on drug titration (cf. Figure 1) **(B)**. The PC combination often does not elicit decrease in SI (A and B). Such "resistant" cases show signs of S-phase arrest as judged from cell cycle distribution histograms **(C)**. %T = proportion of tumor cells, SYN = synergistic effects of the involved drugs; ANT = antagonistic effect of combining drugs; inhibited = complete block of S-phase entry; sub-G1 = indication of apoptotic bodies in the sub-G1 region.
